# Epidemiology of Newcastle disease in Africa with emphasis on Côte d’Ivoire: A review

**DOI:** 10.14202/vetworld.2021.1727-1740

**Published:** 2021-07-03

**Authors:** Charlie Franck Arthur N’Guessan Amoia, Pius Ajanwachukwu Nnadi, Chuka Ezema, Emmanuel Couacy-Hymann

**Affiliations:** 1 Department of Animal Health and Production, Faculty of Veterinary Medicine, University of Nigeria, Nsukka, Enugu State, Nigeria; 2 LANADA/Central Laboratory for Animal Diseases, B.P 206 Bingerville, Côte d’Ivoire

**Keywords:** control, ethnoveterinary medicine, Newcastle disease, prevalence, socio-economic impacts

## Abstract

For decades, Newcastle disease (ND) has long been recognized as a frontline viral disease that constrains poultry production throughout Africa. The need to update on the epidemiology of the disease is rife, due to the increasing importance of poultry farming. In addition, poultry farming serves as the top animal food source globally. However, in Africa, the greater population of poultry is reared under traditional and conventional husbandry methods. This hugely impedes the ability of management practices to be correctly embraced in limiting or excluding viral pathogens in the poultry production chain. We conducted this review to consolidate recently published studies in the field and provide an overview of the disease. We reviewed original studies conducted on ND, the current taxonomic classification of the virus, clinical signs of the disease, and laboratory diagnostic methods available for virus detection and typing. This review additionally examined the control methods currently used, including available or circulating vaccines, vaccinations, recent vaccine findings, and the main variants of the virus present in West Africa. More specifically, we present a review of the current status and available information on the disease in Côte d’Ivoire. The lack of up-to-date and relevant information on the current prevalence, socio-economic impact, and ethnoveterinary medicine used against ND is probably the main limitation for appropriate and effective decision-making for better control of this disease in Côte d’Ivoire.

## Introduction

Over the past two decades, there has been an upward growth trajectory in the poultry subsector by 76% in developing countries compared with 23% in developed countries [1]. In sub-Saharan Africa, the poultry sector is irrefutably rapidly expanding. Principally, poultry meat serves as the most important source of animal protein. Notably, poultry production is practiced in two principal modes: The commercial poultry approach with elaborate facilities, excellent veterinary health-care, and good nutrition, and the village backyard poultry approach where the minimum investment in housing, feeding, and health-care delivery is the norm. Although the importance of poultry contribution in terms of income and protein intake in a developed economy varies widely from that in a developing economy [2], its impact on developing countries is significant since a larger majority of the population is agrarian and rural. In most developing countries, such as Côte d’Ivoire, where eggs and poultry meat are the main sources of dietary protein [3], village chicken (VC) farming plays a pivotal role in the quest for self-sufficiency and sustainable food security. It immensely contributes to the religious, social, economic, and cultural well-being of the rural population [4]. Inarguably, VC production is a major source of income for poor rural farmers, especially women and children, who constitute the major players in the industry. In addition to being a gateway out of poverty for rural women, VCs’ fecal droppings serve as an organic fertilizer for crop agriculture [1].

In Côte d’Ivoire, the contribution of poultry to overall meat production cannot be underestimated. It accounts for approximately 44% of the total national meat production [5] and includes imported broiler and layer birds alongside VC/backyard poultry, commonly referred to as “running” chickens. VC farming is widely practiced by rural populations. According to the Ministry of Animal and Halieutic Resources of Côte d’Ivoire [5], backyard poultry flocks constitute approximately 75% of the national flock with a population of approximately 25,000,000 poultry in 2013. Primarily, chickens are reared mainly for self/home consumption, traditional rituals, donations, and offerings on occasions and festivities, such as marriage and initiatory rites (entry to sacred wood), or to seek favors among members of a population [6]. In the case of social bonding, it is recommended to offer chickens to a person to convey gratitude or as a token of appreciation for a favor or help granted (e.g., to a person holding an official position). In instances where poultry rearing is not the main economic activity of households, the sale of surplus poultry usually generates additional income for owners, which subsequently helps them cope with family health problems, children’s schooling, and/or debts [7]. Approximately 80% of family poultry farming is performed by women and children, who include it in their domestic activities, whereas men are engaged in large-scale farming and other activities. Women and children are often in-charge of distributing grains as food supplements to the chickens. However, men are responsible for social decisions on how the chickens are to be slaughtered or occasions that call for their sales [8]. Most often, more than 89% of small rural households raise chickens with an average of 6.8% [9]. Despite this high average, national poultry production remains deficient. For example, Côte d’Ivoire has imported frozen poultry meat products from African and European countries. These imported animal products amounted to more than $215 million in 2013 [5]. The deficits are attributable to several problems, such as outbreaks of epizootics, infectious pathogens, parasitic infestations, and losses due to predators and nutritional imbalance. However, most VCs are raised without food supplements, vaccination, prophylactic medication, or treatment for recurring ailments. It should also be added that VC farming in Côte d’Ivoire has neither benefited from support programs set up by the government or donor agencies. The few programs that are actively involved in the rearing of local species principally use chickens for improving poultry strains for crossbreeding, particularly for meat production [10]. In this regard, most programs have failed due to the lack of monitoring mechanisms [8]. Moreover, crossbreeding with exotic breeds is a source of extinction of local breeds by leaving hybrids in place, which is not always accessible to the breeder in rural areas [11]. VC farming is practiced throughout the country with a high concentration in the northern part, with an average of at least 1000 poultry per village. The Savannah region (north) provides 40% of the poultry population in the family sector, whereas the Zanzan region (northwest) provides 30% [12]. VC farming has experienced a spectacular boom in the past 3 years following the outbreak of the Ebola virus [13]. Abruptly, all consumers of bush meat suddenly changed their preference to passionately patronize VCs. The situation has made the population to understand that developing the local poultry industry could provide an alternative solution to the fight against poaching and zoonoses and, above all, a realistic means of fighting poverty. Furthermore, promoting family poultry farming could help curb the degradation of the remaining biodiversity and genetic composition of poultry in Côte d’Ivoire. VC production is hampered by Newcastle disease (ND) [14], which is a major constraint to poultry production in Africa and Asia. Concerted efforts are being made to combat this animal disease, as it significantly limits the performance of the poultry subsector [15]. In some parts of Africa, [16] the lack of an adequate control program has resulted in high morbidity and mortality rates among poultry flocks. In VC farming, ND is endemic in all West African Economic and Monetary Union (WAEMU), with epizootic peaks occurring either between November and February or July and August annually, more so during the rainy season. Other peaks can also be observed, particularly during larger poultry movements, for example, at the eve of traditional festivals during which the demand for poultry meat increases [17]. ND is caused by *Avian orthoavulavirus 1* (formerly designated as ND virus [NDV]), which belongs to the genus *Orthoavulavirus* in the family *Paramyxoviridae* under the order *Mononegavirales* [18]. NDV often camouflages itself in respiratory and neurological signs that eventually result in high mortality in chicken populations in and around Africa. The severity depends on the viral factors (tropism and virulence), host factors (age, species, and immune status), and environmental factors (temperature, season, rainfall pattern, and relative humidity) [19]. In ND outbreaks involving the velogenic strain, the morbidity and mortality rates are almost 100% [20]. In rural areas, the disease can kill up to 80% of susceptible poultry and is, therefore, one of the greatest constraints to local poultry production. Hence, the velogenic strain itself solely poses a huge impediment to rural development. Furthermore, difficulties in ensuring cold chain during vaccine transport, failing vaccination programs, and high costs of booster vaccination are few factors limiting the control of this disease [21].

This study was conducted to provide a critical review of the available literature to provide current epidemiological knowledge and determine the effects of ND on local chickens in Africa, with a special focus on Côte d’Ivoire.

## Methodology

The working methodology consisted of a broad review of the available scientific studies on the research topic. Specifically, the literature search focused on search terms, including “VC farming ­systems,” “ND in VC farming,” and “ND control methods in VC farming and its economic impact.” A synthesis of this information was made to obtain the data presented below.

### History of NDV

Historically, the disease was first reported in Indonesia in 1926 [22]. The avian bug got its name from the city of Newcastle-on-Tyne in England, where an outbreak occurred in 1927 [23]. Interestingly, this disease was individualized by Doyle, who in England, described a deadly chicken illness in a nearby farm in Newcastle where 700 adult chickens and chicks of varying ages died. Doyle has named the disease “ND” [23]. The disease is similar to the bird plague described by Centanni in Italy in 1901. However, according to Doyle’s research in 1927, ND differs from avian influenza not only in the causative virus but also in the length of the incubation period. In addition, Doyle has highlighted that contagion and respiratory signs are much more intense in ND. He has also shown that chickens immune to this disease are not protected against the avian influenza virus. Therefore, this condition was reported by several authors and called various names, including *Ranikhet disease, Philippine fowl disease, pseudo-fowl plate, Doyle disease, Korean chicken disease, pseudo-bird plague*, and *Asian bird plague*. At present, the name “ND” is used because of the region where it was first described.

### NDV

ND is caused by strains of *paramyxovirus type 1* (APMV-1), which is characterized by a helically symmetrical capsid and a single-stranded, unsegmented RNA of negative polarity [3]. NDV is an enveloped virus that is part of the recently described genus *Avulavirus* belonging to the order *Mononegavirales*, the family *Paramyxoviridae*, and the subfamily *Avulavirinae* [24]. NDV, formerly known as *Avian paramyxovirus 1* or *Avian avulavirus 1*, was formally known as *Avian orthoavulavirus 1* since 2018. The virus exists in 20 serotypes, APMV-1 to APMV-20 [25], but all NDV isolates belong to serotype 1 (APMV-1). NDV is infective for almost all avian species, both domestic and wild. At present, NDV affects at least 250 species of birds in 27 orders (e.g., chickens, turkeys, ducks and geese, pigeons, peafowl, guinea fowl, pheasants and quail, canaries, psittacines, ratites, and wild waterfowl) [26]. Based on the severity of the disease in chickens, NDV is categorized into asymptomatic enteric (avirulence), lentogenic (low virulence), mesogenic (intermediate virulence), and velogenic (high virulence) pathotypes [27]. NDV strains are characterized by a diversity of their genetic material and by the continuous development of these variabilities [20]. Two taxonomic systems are used. The first concerns the separation of NDV strains into two classes of several lines: Class I includes avirulent strains and Class II consists of virulent and avirulent vaccine strains, such as LaSota and Hitchner B1, both of which are used worldwide [28]. An additional lineage and seven more sub-lineages were later proposed [29]. The conflicts and confusion generated by these two classification schemes necessitated the development of unified criteria for the taxonomy of NDVs. Thus, Diel *et al*. [20] have proposed adopting a genotype-based classification. To date, this classification is the most widely used and gives a stronger correlation between the evolutionary distances of intergenetic groups and their phylogenetic relationships.

### Clinical Manifestations of ND

The clinical signs of NDV infection are strongly influenced by several factors, including virulence and tissue tropism of the virus, species, age, immune status, and condition of the bird. In addition, these clinical symptoms can be influenced by the route of exposure, magnitude of the infectious dose, and underlying external factors, such as housing type and environmental and social stress. Nevertheless, clinical ND is widely classified into four syndromes based on the disease in domestic chickens (Table-1) [30]. In adult poultry, a marked decrease in egg production (egg deposition) may be the first obvious sign, followed by significant mortality [31]. However, according to Ewies *et al*. [28], some strains of NDV cause mild clinical signs, whereas other strains cause acute signs. Based on clinical signs and common lesions, Beard and Hanson [32] have grouped virulent strains into five pathotypes (or disease groups) as follows: (1) Asymptomatic enteric form: The virus presents a subclinical enteric infection without clear symptoms; (2) lentogenic: The virus presents with mild (slightly virulent) respiratory infections; (3) mesogenic: The virus presents with rare nervous and respiratory signs while the mortality rate is related to the age of susceptible birds (young birds are more susceptible than adults); (4) viscerotropic velogenic: The virus causes hemorrhagic intestinal lesions (highly virulent); and (5) neurotropic velogenic: The virus causes high mortality following respiratory and nervous signs (very highly virulent) [27].

**Table-1:** Pathology observed in poultry during infection with Newcastle disease virus [30].

Dominant symptoms	Pathogenicity				

	Velogen		Mesogen	Lentogen	Asymptomatic Enterotropic

	Viscerotropic	Neurotropic			
Diarrhea	+++	−	−	−	−
Respiratory distress	−	+++	++	+	−
CNS syndrome	(++)	+++	(++)	−	−
Fall of the egg laying	+++	+++	++	(+)	−
Morbidity	+++	+++	++	(+)	−
Mortality	+++	++	++	(+)	−

Severity of symptoms observed: +++: strong; ++: intermediate; +: mild; Clinical signs observed only in young animals are indicated in brackets. CNS=Central nervous system

NDV isolates are differentiated based on an *in vivo* estimate of pathogenicity (Table-2). These *in vivo* tests are the meantime of death (mean death time [MDT]) in specific pathogen-free (SPF) embryonated chicken eggs, the intracerebral pathogenicity index (ICPI) in SPF day-old chicks, and the intravenous pathogenicity index (IVPI) in SPF 6-week-old chicks [33].

**Table-2:** Correlation between *in vivo* and *in vitro* methods of characterizing NDV strains.

Test *in vitro*	Pathogenicity		

	Velogen	Mesogen	Lentogen/Asymptomatic
ICPI^a^	>1.5	0.7-1.5	<0.7
IVPI^b^	>2.5	<2.5	<2.5
MDT^c^	<60 h	60-90 h	>90 h
Formation of plates^d^	Yes	Yes	No
Thermostability^e^	Yes	Yes	Yes/No
Cleavage site of the F0 precursor protein	^112^R R/K Q/K R/K R ↓ F^117^	^112^R R/K Q/K R/K R ↓ F^117^	^112^G R Q G R ↓ L^117^

a The Intracerebral Pathogenicity Index (ICPI) is calculated after intracerebral infection of chicks at the age of

one day; A score (0: normal; 1: sick; 2: dead) is assigned to each chick daily for 8 days.

b The Intravenous Pathogenicity Index (IVPI) is calculated ina similar way to the ICPI but in poultry infected intravenously at six weeks of age.

c The mean death time is the average time in hours required to obtain the death of all inoculated embryos.

d NDV induces the formation of plaques on embryonic fibroblast culture whose size and morphology vary according to thevirulence of the viral strain; lentogenic strains require the addition of trypsin in the culture medium. ^e^Infectivity measurement after treatment at 56°C for 5 min (thermostability).

f The lentogenic strains used in vaccination are heat sensitive except for the vaccine strains V4 and I-2; the lentogenic strains isolated from wild animals are heat stable

Using MDT, NDV strains are classified into the following groups: Velogenic (causes death in <60 h), mesogenic (causes death in 60-90 h), and lentogenic (causes death in more than 90 h). The ICPI in day-old chicks is the formal standard virulence test [3]. According to European Directive 92/66/EEC, NDV strains with an average ICPI of >0.7 are considered virulent [3]. The IVPI classifies NDV strains as lentogen and velogen. Slow-growing and some mesogenic strains have IVPI values of 0.0, whereas the maximum IVPI value for a virulent strain is 3.0 [34]. The ICPI classifies NDV strains by giving indices ranging from 0.0 to 2.0. The maximum score of 2.0 is assigned to the most virulent strain of NDV, whereas scores close to 0.0 are assigned to lentogenic strains.

### Status of ND in the African Poultry Subsector

The major impediments to traditional poultry production include endemicity of infectious diseases, predation, lack of proper health-care and biosecurity, poor feeding, and poor marketing information [35]. Most African countries face challenges with diseases in traditional livestock farming. According to Kondombo *et al*. [36], chick losses are caused by ­infectious diseases, among which ND accounts for 83%. According to Halima *et al*. [37], ND and predators are the main causes of chicken losses in Northwestern Ethiopia. Similarly, Aboe *et al*. [38] have also shown that in Benin, Burkina Faso, Mali, Ghana, and Guinea, ND is the major cause of mortality and morbidity in poultry. Several studies have shown the endemicity of this disease in traditional poultry farming in countries, such as Côte d’Ivoire [39,40], Nigeria [41,42], Sudan [43], Uganda [44], Mauritania [45], Niger [46], Tanzania [47], Madagascar [48,49], Togo [50], and Congo [51]. This disease, although not the main cause of death in Cameroon [52], Mali [16], Burkina Faso [53], Chad [54], and Zimbabwe [55], is the second cause of death accounting for, on average, 35%. African farmers are often unable to predict outbreaks of the disease, making it difficult for them to embark on preventive control measures [56]. ND is one of the priority diseases of traditional livestock farming [57].

## Epidemiology of ND in Africa

### Variable genotypes involved in ND outbreaks

ND is endemic in Africa and in other parts of the world, except for Canada, the United States of America, and some Western European countries, where the disease is currently under control [58]. Moreover, since wild birds can sometimes carry the virus without contracting the disease, outbreaks can occur wherever poultry farms are located [59]. The distribution of NDV genotypes is not related to geographical areas. Thus, several genotypes are found in America, Europe, Asia, and Africa. Genotype II is the most widespread in these continents [20]. Two genotypes are frequently described in Africa: Genotypes V and VII [50]. Note that genotype VII isolates are responsible for the fourth and fifth panzootic of ND [60]. Besides, their recent emergence has been reported in some African countries [28]. Snoeck *et al*. [61] have reported high genetic diversity among NDV strains circulating in poultry populations of West and Central Africa and two newly defined genotypes — genotypes XVII and XVIII — based on the analysis of the sequence of the fusion gene (F) (Table-3) [62]. After demonstrating that the average evolution distance between these two genotypes was <10%, knowing that this is the minimum recommended distance to define a new genotype [20], it is speculated that NDV genotypes XVII and XVIII can be considered a single genotype, namely, the XVII genotype [63]. All main variants present in West Africa have been grouped into three genotypes, all belonging to Class II: Genotypes XIV, XVII, and XVIII [61]. Village poultry farming is confronted with ND in three characteristic epidemiological aspects: The epizootic form, the enzootic form, and the seasonal nature of the epizootic diseases [64]. Thus, reclassifying the strains of NDV present in different regions of Africa is necessary. This will help design a more effective control strategy using vaccines matched to genotypes because of the large evolutionary discrepancy between commonly used vaccines and NDV strains currently in circulation.

**Table-3:** Current classification and distribution of class II Newcastle disease virus genotypes [62].

Genotypes	Subgenotypes	Geographic distribution	Remarks
I	Ia, Ib, Ic	Australia, Africa, Europe, US, Asia	Low virulence, Ulster, V4
II	-	North and South America, Africa, Asia, and Europe	Avirulent, lentogenic, Lasota, B1
III	-	Japan and Australia, Taiwan, Zimbabwe	Ancient strains but still emerging, mesogenic Mukteshwar
IV	-	Europe, Africa, Asia	Virulent, Herts/33 (UK)
V	Va, Vb, Vc, Vd	South America, Europe, and Africa	Virulent, Anhinga (US)
VI	VIa, VIb, VIc, VId, VIe, VIf, VIg, VIh,	Europe, Asia, Africa, South America	Pigeon paramyxoviruses
VII	VIIa, VIIb, VIIc,, VIId, VIIe, VIIf, VIIg, VIIh, VIIi	Emerged in Far East in 1990, spread to Europe and Asia, Africa.	Virulent, 4^th^ Newcastle disease panzootic virus, 5^th^ panzootic virus
VIII	-	South Africa, Asia	Highly virulent, AF22440
IX	-	First isolated in China in 1948	Highly virulent
X	-	Taiwan, Argentina, USA	Virulent
XI	-	Madagascar	Virulent, restricted distribution
XII	-	South America and China	Virulent
XIII	XIIIa, XIIIb, XIIIc	Asia, Europe, and Africa	Virulent, continuously emerging
XIV	XIVa, XIVb	West Africa	Highly virulent, recovered from domestic birds only
XV	-	China	Originated from mixed virulent and vaccine viruses
XVI	-	Europe in the 1940s, Africa and Asia in 1980s	Highly related to genotype IV
XVII	XVIIa, XVIIb	West and Central Africa	Highly virulent, continuously emerging evolving
XVIII	XVIIIa, XVIIIb	West Africa	Highly virulent

## Retention, Distribution, and Transmission of NDV in Villages

**Figure-1:**
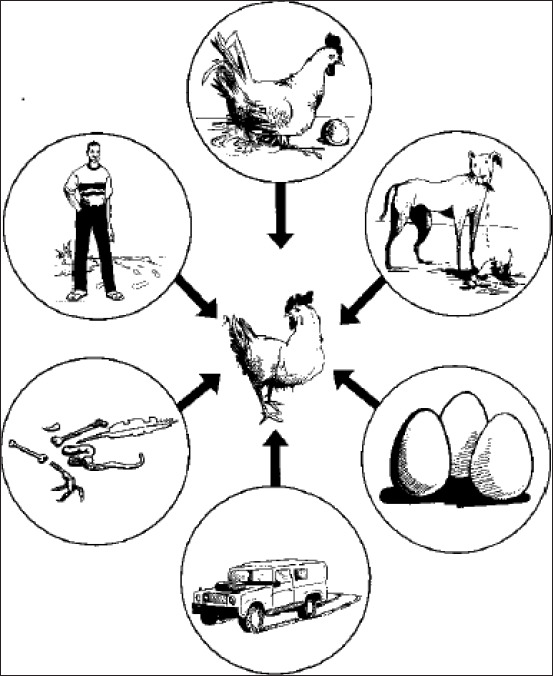
Transmission of ND from one village to another through people, fomites, animals, and their products [72].

Several factors account for the origin, maintenance, and spread of NDV. These include high poultry population, keeping of mixed species of poultry, widespread presence of wild birds, environmental factors, and poultry trade [65]. The heterogeneity of bird populations in family farms places them at greater risk of contracting NDV according to Capua *et al*. [66]. Raising different bird species in the same place can promote the transmission of the virus because some species can harbor the virus without showing any clinical signs, called carriers. This is the case with ducks, which, in mixed farming systems, can infect susceptible chickens [67]. In addition, wild birds contribute to the maintenance of the virus since lentogenic NDVs are found in migratory birds [68]. These viruses can evolve and become virulent after passage in domestic poultry [69]. In a study on active NDV surveillance in wild pigeons (*Columba livia)* in urban population, Ayala *et al*. [70] have demonstrated the presence of vaccine-derived NDVs from 17 species of wild birds across four continents from 1997 to 2014. Other non-avian animals, such as cats, dogs, and rodents, excrete the virus for a short time (72 h) in their feces after eating contaminated poultry. This shows that they can play a substantial role in the spread of the virus through contact with poultry or environmental contamination [48]. In a study on factors associated with ND outbreaks in indigenous VCs in Kenya, Njagi *et al*. [71] have shown that the warm season, low rainfall, savannah-type environment, and medium altitude (760 m) are favorable conditions for the growth and spread of NDV. Poultry traders may also be responsible for the spread of NDV as farmers tend to place suspected poultry and other animals in the market. Several modes of transmission of NDV are known, including transmission through direct contact between healthy birds and infected or carrier birds, contact with the secretion and excretion of infected birds, and contact with contaminated materials (Figure-1) [72]. Another important transmission route is through aerosol [73]. Fleas, rodents, insects, and dogs can also transmit NDV from infected feces [72]. The introduction of infected birds into a susceptible flock results in infection of the flock within 2-6 days [74]. Rigorous implementation of biosecurity measures is an important strategy in controlling ND.

### Spatiotemporal Distribution of ND

**Figure-2:**
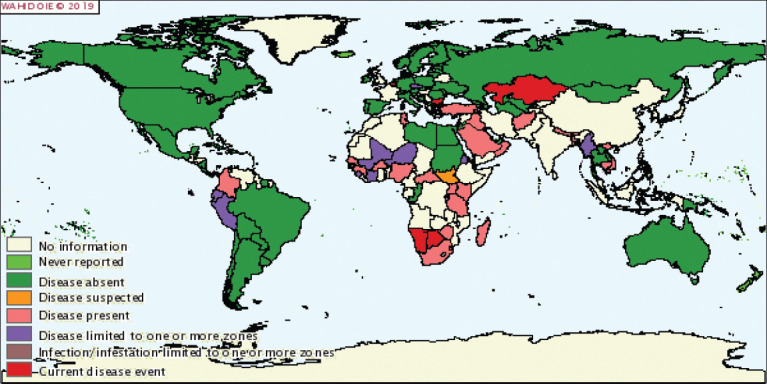
Map showing geographical distribution of Newcastle disease worldwide [59].

Since its appearance in Newcastle-on-Tyne, the disease has spread quickly throughout the world. At present, ND is present in all continents: Europe, Asia, Africa, and America. The continuous changes in the distribution of ND require the World Organization for Animal Health to provide weekly information on the disease and produce a biannual map of the situation of World Animal Health. Figure-2 [59] illustrates the distribution of ND worldwide based on the data provided by the veterinary service departments of individual countries [59]. Referring to the map, it is evident that ND is endemic in Côte d’Ivoire, similar to that in ­several African countries.

### Control of ND

**Figure-3:**
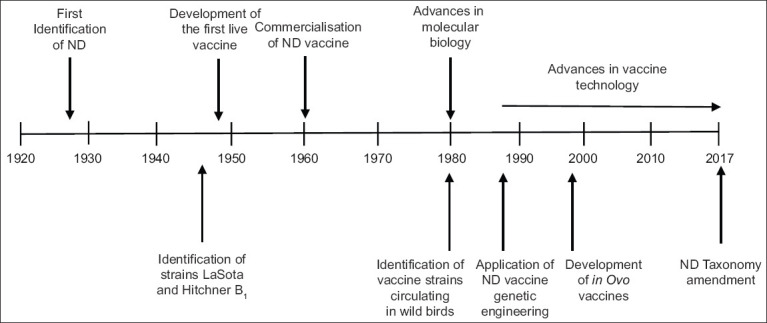
Schematic summary of the timeline of Newcastle disease vaccine development [21].

Vaccination is the main method for controlling ND [64]. Vaccination against ND in domestic poultry was first proposed in the early 1930s, shortly after the identification of NDV [22,23]. Figure-3 [21] shows a timeline of the evolution of ND vaccination since the 1930s.

Vaccination against NDV dates back more than 60 years. Vaccine-induced immunity is short-lived and currently considered to past 10-12 weeks. To maintain adequate protection, repeated vaccinations are needed. Notably, parental immunity also interferes with vaccine effectiveness. Therefore, vaccination programs are often delayed until chicks reach the age of 1-2 weeks. Two type of vaccines are predominant: Inactivated and live vaccines [75]. Inactivated vaccines are considerably more expensive compared with live vaccines due to the need to handle individual birds and provide an injection, either intramuscularly or subcutaneously [70]. Live vaccines are administered by eye drops, in drinking water, by aerosol, or intranasally. Both live and inactivated vaccines are produced using SPF embryonated eggs [75]. The advantages of live vaccines include their relative cheapness, stimulation of local immunity, ease of application through mass medication, and their ability to confer protection soon after vaccination. Conversely, the disadvantage of lentogenic virus vaccines is their potential capacity to induce disease in susceptible chicks and their ability to mimic infections, such as infectious bronchitis and other respiratory infections [76]. Consequently, low pathogenic strains of the virus are used for the initial vaccination, which, in turn, requires revaccinations. The efficacy of lentogenic virus vaccines depends on the ability of the virus to multiply in chickens and stimulate immunity, particularly in the face of maternal immunity. The virus’ ability to spread from bird to bird is also important in exposing all birds to infection.

Oil-based, inactivated vaccines are widely used and usually injected intramuscularly. These vaccines have been used in areas where ND is endemic to revaccinate laying and breeding birds previously vaccinated with a lentogenic dose. Despite the availability of effective vaccines from large pharmaceutical companies, they are rarely used in environments with high ambient temperatures, poor infrastructure for vaccine storage and transportation, small-scale poultry operations, and a shortage of foreign exchange [77]. Several studies have shown that *Avian Avulavirus* Type 1 strains continue to evolve and have been associated with vaccine failures [78]. Vaccination continues to play a pivotal role in controlling and eradicating ND worldwide. As shown in Table-4 [21], the most commonly used vaccine strains are LaSota and Hitchner B1 (lentogenic strains), which remain the precursor for most vaccines currently available in the market [21].

**Table-4:** Summary of the main AAvV-1 strains utilized in current Newcastle disease vaccine production [21].

Virus strain	Pathotype	Vaccine type	Origin
F	Lentogenic	Live or inactivated	India
Hitchner B1	Lentogenic	Live or inactivated	USA
LaSota	Lentogenic	Live or inactivated	USA
V4	Avirulent	Live or inactivated	Australia
V4-HR	Avirulent	Live (Thermostable)	Australia
I-2	Avirulent	Live (Thermostable)	Australia
Mukteswar	Mesogenic	Live (Booster)	India
Komarov	Mesogenic	Live (Booster)	Israel

Advances in vaccine technology have allowed the development and use of novel strategies to help improve vaccine efficacy. This is seen in the evolution of DNA vaccines [79], recombinant vaccines [80], vaccine-coated cereal grains [81], and multiplexed vaccines offering protection against multiple pathogens within a single immunization [82]. The simultaneous application of biosecurity measures, quarantine of new animals, rigorous farm management, and vaccination are certainly the best management systems in the fight against ND.

### ND as a Zoonosis

In addition to avian species, humans are among several species that can be infected with NDV. In humans, exposure to large amounts of NDV causes conjunctivitis and/or influenza-such as symptoms, including fever, headache, and malaise [83]. Laboratory workers, vaccinators, and veterinarians are the most susceptible. The use of personal protective equipment and biological safety cabinets has reduced the exposure of laboratory personnel to this virus. Infection is rarely observed among farm staff. People handling or consuming poultry products do not appear to be at risk [84]. Conjunctivitis usually disappears quickly, but the virus will be released into the eye and then discharges in 4-7 days. In addition, mild and self-limiting cases of influenza, such as fever and headaches, have also been reported in humans [27]. No evidence of human-to-human transmission has been found, but the potential for human-to-bird transmission exists [27]. The risk of NDV transmission from animals to humans and vice versa is high in backyard poultry flocks due to the proximity between poultry and humans and the low application of quarantine, biosecurity measures, and vaccination against NDV. Special precautions must be taken for immunocompromised individuals who work closely with domestic poultry or for poultry workers who live with immunocompromised individuals [85].

### Ethnoveterinary Medicine in the fight against ND

It is a common experience these days that antimicrobial agents could face diminishing efficacy after some periods of use [86]. In addition, there are growing concerns over chemical or antimicrobial residues in animal products meant for human consumption [87]. These have created the need for alternative approaches in addressing microbial scourge in humans and animals. At present, ethnomedicine and ethnoveterinary medicine, which have cultural roots in many traditional societies before modern chemotherapy, are being recognized and exploited as adjuncts to or replacements of modern microbial cure. In these alternative approaches to microbial cure or containment, natural products that leave no residues in animal products and, therefore, pose no risks to consumers are used. The interest of stakeholders in veterinary health delivery began to shift toward ethnoveterinary science in the early 1980s. The pursuit of the incorporation of ethnoveterinary practice into modern veterinary practice started by defining two main objectives: Identifying traditional veterinary health-care practices and documenting them to have a written record of “what” and “how” for posterity and advancing these practices before they are lost, given that these practices are often transmitted from generation to generation verbally [88]. These are also being revived in developed countries, where awareness is being raised at both the consumer and farmer levels [89]. Some age-long practices have been identified where herbs and herbal mixtures have been identified as effective means of antimicrobial treatment in animals. Some studies exist that confirm the effectiveness of some traditional remedies used by farmers against ND. Khan *et al*. [90] have demonstrated that the addition of dried garlic powder (*Allium sativum*) into poultry rations increases post-vaccination antibody levels against NDV, infectious bronchitis, and Gumboro disease. In another study, Omolade and Hauwa [91] have shown that only anti-NDV antibodies are increased. In studies conducted on broilers and laying hens, the addition of neem (*Azadirachta indica)* leaves in drinking water increased the post-vaccination antibody titers of animals, while increasing the overall growth performance [92,93]. Nwude and Ibrahim [94] have shown that ND can be treated by soaking *Lagenaria vulgaris* in water and subjecting flock members to drink the extract.

Another method is soaking the bark of *Parkia filicoidea* for the same extraction overnight in water, which is later made available to the chicks. According to McCorkle and Mathias-Mundy [95], the disease can also be treated by water extraction of the bark of *Cassia didymobotrya* or *Euphorbia matabelensis* latex and making the water extract available to the chicks. According to Alders and Spradbrow [60], other traditional remedies are administered to animals to control and treat microbial infections. These include water extracts of chili pepper, mango bark, and white vinegar and garlic seeds mixed with corn bran. Furthermore, cactus sap mixed with corn bran and water, peppers and garlic in water, and mango leaves crushed in drinking water are other forms of traditional remedies. In a study in Togo, Yao *et al*. [96] have shown that some farmers use 1-kg *Khaya Senegalensis*, *P. suberosa*, or *E. poissonii* concoction, which they mixed with 5-L water before serving the mixture to poultry. The most used part of the plant is the root, and the least used part is the wood [97]. Table-5 [10,89,94,95,98-105] shows some remedies used against ND in traditional settings.

**Table-5:** Selection of the most interesting traditional remedies used in the prevention of Newcastle disease [89].

Name of the plant	Part used	Preparation	Administration	Posology	Reference and country
*Allium sativum*	Clove	Syrup to keep for two weeks in the shade before use	Oral in drinking water	Every day, either only during the dry season or all year round	[89] Cambodia
*Zingiber officinale* Roscoe	Rhizome				[89] Cambodia
*Allium sativum*	Clove	Syrup	Oral in food	All the days	[89] Cambodia
*Zingiber officinale* Roscoe	Rhizome	Dye to keep for three weeks before use	Oral	One drop when the chicken is 2 days old, three drops when the chicken reaches 1 kilo	[89] Cambodia
*Capsicum annuum L.*	Fruit				[89] Cambodia
*Alpinia galanga* (L.) Willd	Leaf				[89] Cambodia
*Piper nigrum L*	Bay				[89] Cambodia
*Alpinia galanga* (L.) Willd	Leaf				[89] Cambodia
*Allium sativum*	Clove	Aqueous maceration	Oral in drinking water	All the days	[89] Cambodia
*Cymbopogon citratus (DC). Stapf*	Whole plant				[89] Cambodia
*Azadirachta indica* A Juss	Bark				[89] Cambodia
*Ceiba pentandra* (L.) gaertn	Bark				[89] Cambodia
Parkia filicoidea	Bark		Put into drinking water		[94] Nigeria
*Cassia didymobotrya* or	Leaves		Added to drinking water		[95] Zimbabwe
*Euphorbia matabelensis*	latex		Added to drinking water		[95] Zimbabwe
*Fruit of Capsicum annuum together with* leaves of *boza multiflora*	Fruit and leaves				[98] Tanzania
Fruits of *Lagenaria breviflora* and v *Capsicum frutescens*	Fruits and frutescens		Put into drinking water		[10] Nigeria
Bark of *Khaya senegalensis* and *Capsicurn* sp. extracts	Bark and extracts		Soaked in drinking water		[99] Senegal
*Mangifera indica*	Barks		Put into drinking water		[100] Gambia
*Mucuna* sp.	Leaves		Crushed leaves soaked in drinking water		[101] Kenya
Barks of *Combretum micranthum + Butyrospermum parkii + Ficus* sp.	Barks		Dried, ground and soaked in drinking water		[102] Burkina Faso
*Lannea acida*	*Barks*		Soaked in drinking water		[101] Burkina Faso
Cassia sieberiana	*Barks*		Used as infusion		[103] Mali
Hot pepper, elephant feces, sisal leaves and leaves from plants known as *“chunga,” “hunduhundu,” and “mwambalasimb”*					[104] Tanzania
*Solanum nodiflorum*			Cut into two halves and soaked in water		[105] Nigeria
*Khaya senegalensis*			Soak in water		[105] Nigeria
*Vernonia amygdalina*			Soak the bark in water		[105] Nigeria
*Datura metel*			Soak in water		[105] Nigeria
*Solanum nodiflorum*			Soak in water		[105] Nigeria
*Capsicum* spp.			Soak in water		[105] Nigeria
*Khaya senegalensis*			Soak in water		[105] Nigeria
*Khaya senegalensis*			Soak in water		[105] Nigeria
*Solanum nodiflorum*			Soak in water		[105] Nigeria
*Vernonia amygdalina*			Soak in water		[105] Nigeria
*Datura metel*			Soak in water		[105] Nigeria
*Monosodium glutamate*			Soak in water		[105] Nigeria

## The Case of Côte D’ivoire

### ND in Côte d’Ivoire

Backyard poultry production faces many disease challenges. The major pathologies encountered in VC farming in Côte d’Ivoire are ND, parasitic infestations, and, since 2006, highly pathogenic avian influenza [39,106]. Annual outbreaks of ND are mainly observed during the rainy season (July to August) and during the dry season (December to February), which is the peak period [39]. From 2007 to 2009, active surveillance was performed in backyard poultry flocks (chickens, guinea fowls, and ducks) in four areas of Côte d’Ivoire. The result has indicated that of the 2680 sera processed, 531 (19.8%) were positive for ND, confirming the endemic presence of ND in Côte d’Ivoire (Table-6) [39]. Few owners of outdoor poultry have reported disease outbreaks, and no reporting system exists at the national level. This lack of participatory monitoring is a major impediment to better control of ND in the country.

**Table-6:** Observed clinical signs and serology results for the 2007-2009 surveillance period [39].

Sampling region	Clinical signs	Serological results: number of positive serum samples per year for Newcastle disease virus		

		2007	2008	2009
South	No clinical signs	48 (16-512)	67 (16-256)	59 (16-128)
North	Cough, inappetence, diarrhea (43/180)	50 (16-64)	26 (16-256)	33 (16-256)
East	No clinical signs	85 (16-1.024)	65 (16-64)	42 (16-512)
West	Inappetence, nasal discharge (13/150)	30 (16-1.024)	18 (16-128)	8 (16-128)
Total positive		213/910	176/1.024	142/746

In another study conducted from 2010 to 2012 to assess the prevalence of NDV in Côte d’Ivoire, molecular screening was performed on more than 22,000 avian samples and serological tests were conducted on nearly 2000 avian sera. Results showed that NDV seroprevalence reached 22% (for the 2000 sera), and molecular screening with polymerase chain reaction (PCR) revealed that 14.7% were positive for NDV [40]. These studies, in addition to the aforementioned studies, have indicated the endemic nature of ND in Côte d’Ivoire. In the same epidemiological study, the pathogenicity of NDVs circulating in the country was assessed. The results showed the existence of cyclic and lentogenic viruses on Ivorian soil, with twice as many cyclic viruses as lentogenic viruses [40,50]. All these data suggest a more committed fight against the disease in traditional environments, given the damage that cyclic viruses can cause, as well as a reassessment of the current situation in Côte d’Ivoire.

### ND Control in Côte D’ivoire

In Côte d’Ivoire, no real national poultry improvement or ND vaccination program is in place at the level of national veterinary services. Few programs that are involved in the rearing of local species are used for improving poultry strains for crossbreeding, particularly for meat production [10]. Most programs have failed due to the lack of monitoring [8]. However, some national village ND vaccination campaigns have been supported by some projects funded by the African Development Bank, Food and Agricultural Organization, and the International Atomic Energy Agency [106].

The political crisis in 2002-2011 severely affected the capacities of most actors in the livestock sector in Côte d’Ivoire. To prevent the harmful effects of ND, traditional poultry vaccination was resumed in 2012 in the north of the country. The latter was made possible through a World Bank-funded project known as West Africa Agricultural Productivity Program (WAAPP), which, from 2012 to 2013, in collaboration with the Directorate of Veterinary Services, initiated two traditional poultry vaccination campaigns against ND in the northern zone of Côte d’Ivoire. However, the system set up by WAAPP, which was supposed to enable the veterinary and management services to take over to achieve self-sufficiency in animal proteins in Côte d’Ivoire, has not followed its course [17].

In VC farming, the level of biosecurity is low, if not non-existent, in the on-farm management. The marketing circuit causes many disease control problems, including a lack of application of biosecurity measures. Market vendors do not apply biosecurity measures, whereas slaughter and plucking areas do not meet any standards. Côte d’Ivoire practices VC farming throughout its territory and imports VCs from neighboring countries, particularly Burkina Faso. As a result, chickens produced in the north of the country and imported ones are transported to the country’s major cities, particularly Abidjan (capital), for marketing. Although veterinary inspections and control are performed on the poultry trade at land border crossings, some animals that carry the virus, which may as yet show visible signs of ND at the time of border crossing, may also be allowed to enter the country. These constraints, in addition to the non-housed, roaming management of the village poultry, absence of biosecurity, and lack of quarantine of newly purchased birds, are some factors contributing to the perennial spread of NDV in traditional poultry farming in the country.

### Socio-economic Impact of ND on Poultry Farming in Africa and Côte D’ivoire

In most developing countries, poultry meat is an essential source of protein in people’s diets because it is affordable and accessible [107]. Chicken meat is used as a source of human nutrition and income and in religious observances. The socio-economic contribution of traditional poultry farming helps combat malnutrition and poverty in rural areas. Recent increases in poultry production have been observed in West Africa, especially the commercial poultry setup that comprises broiler and pullet production [108]. Moreover, VCs, which are traditionally managed with limited resources, are predominant and bring added benefits of complimenting family medical care bills, children’s clothing, and school fees. However, around the world, the poultry industry suffers severe economic losses each year due to infectious diseases [109,110].

Poultry diseases reduce poultry production capacity, and related mortality rates can reach as high as 80%. The socio-economic impacts are all the more significant in the presence of avian influenza and ND, which affect all actors in the sector with direct consequences on the producer’s incomes, unemployment, and other affiliated poultry sectors. When production is disrupted by an ND outbreak, the aforementioned services are negatively affected [14] due to high morbidity and mortality. According to Gautier [17], the disease occurs at least annually among village poultry flocks in the WAEMU and potentially affects approximately 135 million animals, representing 80% of the total poultry population. In monetary terms, this loss amounts to approximately $458 million per year. In these countries, where eggs and poultry meat are essential sources of dietary protein, the disease, due to its endemic nature, cripples poultry production. According to Maho *et al*. [14], the economic impact of ND is enormous and classified into the following categories: “Direct losses” (visible losses, e.g., deaths and stunting, or invisible losses, e.g., reduced fertility and changes in herd structure) and “indirect losses” (additional costs, e.g., drugs and vaccines, and forgone revenues, e.g., denied access to better markets). In countries where the virus is endemic, and outbreak is prevalent, control measures, including vaccination, repeated testing, and revaccination helps prevent disease outbreaks. ND control is a major challenge in chicken production in rural areas and presents particular challenges for authorities in developing countries, including Côte d’Ivoire. A study on the real economic impact of ND on VC farming would be an asset for a better estimation of the problem of ND in Côte d’Ivoire.

## Conclusion

The epidemiology of ND in Côte d’Ivoire, as well as its actual economic impact, remains poorly understood. Information on the use of ethnoveterinary medicine in the fight against ND in African countries, particularly in Côte d’Ivoire, is limited. Further studies should also be conducted to determine the genotype(s) and serotype(s) of the viral strains present in the areas for effective vaccination development. Furthermore, a study is needed to bring greater clarity and understanding in the epidemiology of ND and to assess its economic impact and identify the traditional control measures, which, adjunct to vaccination, will improve control options. Such a study will help in decision-making in the fight against ND.

## Authors’ Contributions

CFANA: Conceptualized, designed, collected all relevant publications, and prepared the first version. CFANA, PAN, CE, and EC: Edited and revised the whole manuscript and provided the necessary information to finalize the manuscript. PAN and CE: Copyedited the English version of the manuscript. All authors have read and agreed to the published version of the manuscript.
